# A Single-Food Substitution Strategy (SFSS) Improves Fat Mass and Metabolic Parameters in MASLD: A Prospective Pilot Study

**DOI:** 10.3390/nu18121873

**Published:** 2026-06-10

**Authors:** Nicole Cerabino, Caterina Bonfiglio, Leonilde Bonfrate, Rosanna Donvito, Pasqua Letizia Pesole, Dolores Stabile, Endrit Shahini, Gianluigi Giannelli

**Affiliations:** 1 Center of Nutrition for the Research and the Care of Obesity and Metabolic Diseases, National Institute of Gastroenterology IRCCS “Saverio de Bellis”, 70013 Castellana Grotte, Bari, Italy; nicole.cerabino@irccsdebellis.it (N.C.); leonilde.bonfrate@irccsdebellis.it (L.B.); rosanna.donvito@irccsdebellis.it (R.D.); 2 Unit of Data Science, National Institute of Gastroenterology IRCCS “Saverio de Bellis”, 70013 Castellana Grotte, Bari, Italy; 3 Department of Human Sciences and Promotion of the Quality of Life, San Raffaele Rome University, 00166 Rome, Italy; 4 Core Facility Biobank, National Institute of Gastroenterology IRCCS “Saverio de Bellis”, 70013 Castellana Grotte, Bari, Italy; letizia.pesole@irccsdebellis.it (P.L.P.); dolores.stabile@irccsdebellis.it (D.S.); 5 Department of Gastroenterology, National Institute of Gastroenterology IRCCS “Saverio de Bellis”, 70013 Castellana Grotte, Bari, Italy; endrit.shahini@irccsdebellis.it; 6 Scientific Direction, National Institute of Gastroenterology IRCCS “Saverio de Bellis”, 70013 Castellana Grotte, Bari, Italy; gianluigi.giannelli@irccsdebellis.it

**Keywords:** MASLD, obesity, plant-based diet, cruciferous vegetables, fat mass, body composition, vitamin D, follistatin, dietary intervention, hepatic steatosis

## Abstract

**Background**: Metabolic dysfunction-associated steatotic liver disease (MASLD) is associated with obesity, insulin resistance, and altered body composition. Although dietary intervention is a cornerstone of treatment, complex or calorie-restricted regimens may reduce long-term adherence. This study evaluated the effects of a pragmatic, short-term intervention that involved replacing one daily carbohydrate serving with cruciferous vegetables on body composition and metabolic parameters in individuals with obesity and MASLD. Associations between changes in fat mass and vitamin D and follistatin levels were also explored. **Methods**: In this prospective pilot study, 44 adults with obesity and MASLD followed a two-month intervention, substituting one daily serving of carbohydrate-rich foods with 200 g of cruciferous vegetables, without prescribed caloric restriction. Anthropometric, bioimpedance, biochemical, and FibroScan assessments were performed at baseline and post-intervention. Changes were analyzed using the Wilcoxon signed-rank test, Spearman’s correlation analysis, and generalized estimating equation (GEE) models adjusted for confounding factors. **Results**: The intervention was associated with a significant reduction in fat mass (−4.86 kg, *p* < 0.001), corresponding to an average relative decrease of approximately 12% along with improvements in metabolic and hepatic parameters. Changes in fat mass were inversely correlated with changes in vitamin D (rho = −0.33, *p* = 0.035), fat-free mass (rho = −0.37, *p* = 0.018), and follistatin (rho = −0.24, *p* = 0.143). In multivariate GEE models, the intervention remained independently associated with fat mass reduction (β = −5.190, *p* < 0.001). **Conclusions**: A simple carbohydrate-to-vegetable substitution without prescribed caloric restriction was associated with improvements in body composition and metabolic health. These exploratory findings suggest that pragmatic dietary modifications may provide clinically meaningful metabolic benefits and support the feasibility of minimal dietary substitution strategies in this population. However, causal inferences remain limited by a single-arm pilot design and require confirmation in larger randomized controlled trials.

## 1. Introduction

Metabolic dysfunction-associated steatotic liver disease (MASLD) is currently recognized as the hepatic manifestation of systemic metabolic dysfunction and represents one of the most prevalent chronic liver diseases worldwide, affecting approximately 30–40% of the adult population [1]. MASLD is closely linked to obesity, insulin resistance, dyslipidemia, and chronic low-grade inflammation, all of which contribute to hepatic lipid accumulation and progressive liver injury [2]. Excess fat and increased fat mass (FM) are central to the development of MASLD, acting through lipotoxicity, oxidative stress, and inflammatory pathways [3]. Therefore, reducing adiposity and improving body composition are key therapeutic targets, as even a 5–10% reduction in body weight has been associated with clinically meaningful improvements in hepatic steatosis and metabolic dysfunction [4].

Currently, there is no universally approved pharmacological treatment for MASLD. Instead, lifestyle changes remain the primary approach, with dietary modifications being the most effective non-drug method [5]. Diet-induced weight loss has been shown to significantly improve hepatic fat accumulation, insulin resistance, and cardiometabolic risk factors [6]. Besides caloric restriction, increasing attention is directed toward diet quality and composition, especially dietary patterns rich in plant-based foods [7].

Plant-rich dietary patterns, such as the Mediterranean diet, are linked to better metabolic outcomes, including improved lipid profiles, glycemic control, and liver function in individuals with metabolic disorders, including MASLD [8]. These diets emphasize a high intake of vegetables, fruits, whole grains, legumes, and nuts, which supply dietary fiber, antioxidants, polyphenols, and essential micronutrients that support anti-inflammatory and metabolic regulation [9]. Recent systematic reviews indicate that plant-based foods may improve anthropometric parameters, lipid metabolism, and liver enzyme levels in individuals with MASLD and related metabolic issues [8].

Among plant-derived foods, cruciferous vegetables belonging to the *Brassicaceae* family—such as broccoli, cabbage, cauliflower, and Brussels sprouts—have attracted increasing scientific attention due to their high levels of glucosinolates and different bioactive metabolites, including isothiocyanates and indoles [10]. These compounds, particularly sulforaphane, have been shown to modulate oxidative stress responses, inflammatory pathways, and lipid metabolism [11]. Experimental and epidemiological studies suggest that regular consumption of cruciferous vegetables may reduce the risk of chronic diseases, including cardiovascular disease, metabolic disorders, and certain cancers [11]. In addition, their high fiber content and low energy density may promote satiety and improve metabolic regulation, supporting body weight management and metabolic health [12]. Although cruciferous vegetables were specifically selected because of their high glucosinolate and isothiocyanate content, other plant-derived foods may also exert beneficial metabolic effects through increased fiber intake, antioxidant activity, and modulation of glycemic responses. However, compared with broader plant-based dietary patterns or the Mediterranean diet, the present intervention was designed as a highly pragmatic single-food substitution strategy intended to minimize dietary complexity and maximize adherence in routine clinical practice.

It is well known that diets rich in refined carbohydrates have been associated with increased adiposity, insulin resistance, and hepatic fat accumulation [13]. Conversely, replacing carbohydrate-rich foods with vegetables may reduce the glycemic load while increasing dietary fiber intake and micronutrient density, thereby improving metabolic homeostasis and facilitating weight reduction [14]. Such dietary substitutions may represent a practical and sustainable approach to improving adherence to nutritional interventions in individuals who are overweight or obese.

In addition to dietary composition, several biological factors may influence metabolic responses to dietary interventions and changes in body composition. Vitamin D has increasingly been recognized as a modulator of metabolic health, with roles in adipose tissue regulation, insulin sensitivity, and inflammation [15]. Vitamin D deficiency is highly prevalent among individuals with obesity and MASLD, with studies reporting insufficiency or deficiency rates exceeding 60–80%, significantly higher than those observed in metabolically healthy individuals without obesity [16]. Emerging evidence suggests that vitamin D status may influence body composition and metabolic outcomes during weight loss interventions [17].

Another biomarker of growing interest in metabolic research is follistatin, a glycoprotein belonging to the transforming growth factor-β superfamily that regulates muscle growth, adipose tissue metabolism, and energy homeostasis [18]. Recent studies suggest that circulating follistatin may play a role in metabolic regulation and inflammatory processes in patients with MASLD and obesity [19]. Follistatin has been associated with the regulation of glucose metabolism, insulin sensitivity, adipose tissue remodeling, and inflammatory signaling pathways, suggesting a potential role in the metabolic adaptations occurring during dietary interventions [19].

Therefore, the present study aimed to evaluate the effects of a two-month plant-enriched dietary intervention—consisting of the replacement of one daily portion of carbohydrates with cruciferous vegetables—on fat mass and metabolic parameters in individuals with obesity. Additionally, we investigated the relationships between changes in fat mass and variations in vitamin D, follistatin, and fat-free mass and estimated the longitudinal effect of the dietary intervention after adjustment for relevant demographic and biological confounders.

This approach differs from conventional dietary interventions by isolating the metabolic impact of a single, targeted food substitution, thereby providing insight into the potential of minimal dietary changes to induce clinically meaningful effects.

Understanding whether simple dietary substitutions can produce measurable metabolic benefits represents an important area of nutritional research.

**Figure 1:**
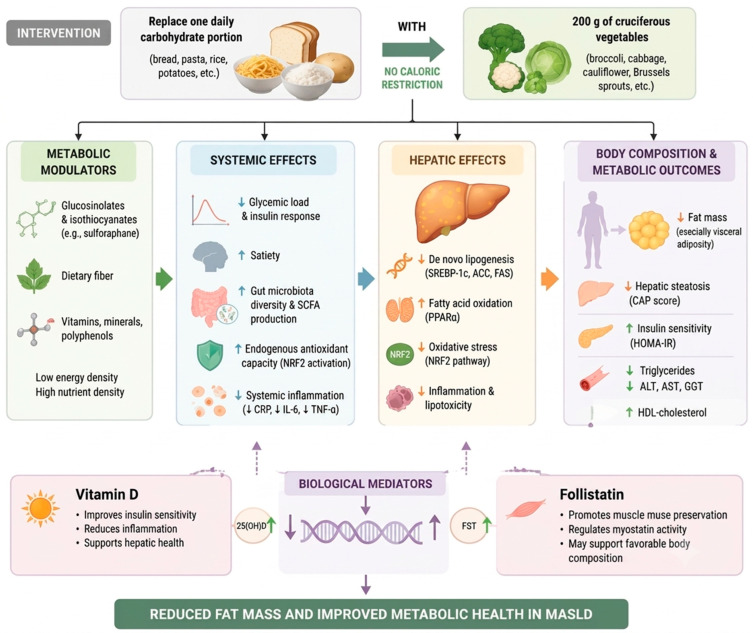
Proposed mechanistic framework of the single-food substitution strategy (SFSS). Replacing one daily portion of carbohydrate-rich foods with 200 g of cruciferous vegetables may reduce the glycemic load and increase the intake of fiber and bioactive compounds (e.g., glucosinolates and isothiocyanates). These changes may modulate systemic metabolism, gut microbiota, and inflammatory pathways, contributing to reduced hepatic lipogenesis, improved lipid oxidation, and decreased oxidative stress. Overall, this process may lead to reduced fat mass and improved metabolic and hepatic parameters in individuals with MASLD. Vitamin D and follistatin are depicted as potential modulators of the metabolic response.

Based on current evidence, we propose a mechanistic framework underlying the metabolic effects of this single-food substitution strategy (Figure 1).

## 2. Materials and Methods

### 2.1. Study Design and Population

This two-month prospective pilot study was carried out by the Clinical Nutrition Research Center for Obesity and Metabolic Diseases at the National Institute of Gastroenterology “Saverio de Bellis” in Castellana Grotte, Bari, Italy. Participants eligible for the study were aged 18 to 65, had a BMI of at least 30 kg/m^2^, and were not taking any medications. Exclusion criteria included diagnosed or newly diagnosed diabetes mellitus, cardiovascular conditions, respiratory failure, severe gastrointestinal diseases, chronic kidney disease (e.g., estimated glomerular filtration rate < 60 mL/min/1.73 m^2^), psychiatric disorders, pregnancy or breastfeeding, eating disorders, non-metabolic chronic liver diseases, alcohol intake exceeding 30 g/day for men or 20 g/day for women, substance abuse, infectious diseases, acute illnesses affecting inflammatory markers, rare metabolic diseases, and mitochondrial fatty acid oxidation disorders.

Participants with obesity underwent anthropometric measurements, bioimpedance analysis, and laboratory tests, including hematological and biochemical parameters. Physical activity was assessed using the International Physical Activity Questionnaire (IPAQ) [20], and adherence to the Mediterranean diet was evaluated with the PREDIMED questionnaire [21]. Smoking habits and medical histories were also recorded. The study protocol was approved by the local Medical Ethics Committee (DDG-CE-502/2005; DDG-CE-792/2014, on 20 May 2005 and 14 February 2014) and conducted in accordance with the ethical standards of the 1964 Declaration of Helsinki. All participants provided written informed consent before enrollment. Recruitment took place from May 2023 to December 2024. Two clinical visits were scheduled: baseline (T0) and after two months (T1). During both visits, data collection included fasting blood samples, anthropometric assessments, and instrumental evaluations : BIA and FibroScan® (Echosens, Paris, France).

Participants were asked to complete a three-day food diary, including two weekdays and one weekend day. After reviewing their typical dietary habits, they were instructed to remove one daily carbohydrate portion, such as bread, pasta, or potatoes, and replace it with a 200 g serving of vegetables. Standard carbohydrate portions were defined according to common Italian dietary serving sizes routinely used in clinical nutritional practice (e.g., approximately 80 g of pasta, 50 g of bread, or 200 g of potatoes). Participants were instructed to replace only one of these habitual portions per day with 200 g of cruciferous vegetables while maintaining the remainder of their usual dietary habits unchanged. No additional lifestyle or diet modifications were suggested. The vegetables included four *Brassicaceae* species: *Brassica rapa* var. *cymosa*, *Cichorium intybus*, *Brassica oleracea* L. var. *sabellica*, and *Sinapis arvensis* var. *orientalis* [22]. These vegetables are known for their high levels of bioactive compounds like isothiocyanates and phenolics, which have demonstrated antioxidant and anti-lipogenic effects in preclinical studies on MASLD [23].

Although total energy intake was not strictly prescribed, participants were instructed to maintain their habitual dietary patterns except for the specified substitution.

Adherence to the intervention was monitored through repeated self-reported three-day food diaries reviewed by trained clinical nutritionists during follow-up visits. Participants were specifically asked to document the daily consumption of cruciferous vegetables and the corresponding carbohydrate substitution. Nevertheless, we acknowledge that self-reported dietary assessment may be subject to recall bias, underreporting, and incomplete recording.

This approach was intended to reflect a real-world, pragmatic dietary modification rather than a tightly controlled feeding protocol.

Importantly, the intervention was designed as a qualitative modification rather than a quantitative reduction in energy intake. Although replacing a carbohydrate portion with vegetables may theoretically reduce caloric intake, this effect was neither quantified nor enforced, and participants were not encouraged to reduce portion sizes or overall food consumption. Therefore, the intervention should be interpreted as a food-based substitution strategy rather than a structured caloric restriction. This design allows for the evaluation of minimal and sustainable dietary modifications that may be more easily implemented in routine clinical practice. However, the potential contribution of unmeasured changes in total energy intake cannot be excluded.

### 2.2. MASLD Assessment

FibroScan is a reliable, cost-effective, and non-invasive method for assessing hepatic steatosis and fibrosis in at-risk individuals [24]. While liver biopsy remains the definitive standard for diagnosing steatosis, fibrosis, and inflammation, ultrasound-based elastography using FibroScan provides a painless, comprehensive liver evaluation and is recommended as a first-line diagnostic tool [25].

Examinations using FibroScan were conducted by skilled operators in accordance with standard clinical practice and current guidelines. Probe selection followed the manufacturer’s instructions, with XL used for BMI ≥ 30 kg/m^2^. Valid measurements required at least 10 successful shots, a success rate of ≥60%, and an IQR/median ratio below 30%, in line with EASL recommendations [5]. Vibration-controlled transient elastography (VCTE) combined with a controlled attenuation parameter (CAP) at 3.5 MHz was employed to estimate liver fat content. CAP thresholds for grading steatosis were 248 dB/m for mild, 268 dB/m for moderate, and 280 dB/m for severe steatosis [5]. Liver stiffness measurement (LSM) was used to evaluate fibrosis, with cutoff values of 8 kPa indicating fibrosis and 12 kPa indicating advanced fibrosis (stage 3).

### 2.3. Anthropometric Parameters

Height and weight were measured in fasting participants wearing light clothing, barefoot, with an empty bladder, and no shoes. BMI was calculated using standardized equipment across all participants. Waist circumference was measured at the midpoint between the lower border of the ribcage and the iliac crest. Although waist circumference was assessed, waist-to-hip ratio was not systematically collected and should be considered in future studies to better characterize visceral adiposity. Systolic and diastolic blood pressure (SBP and DBP) were measured three times with patients seated and resting, using an automatic monitor: OMRON M6 (OMRON Healthcare Co., Ltd., Kyoto, Japan).

### 2.4. Bioelectrical Impedance Analysis (BIA)

Bioelectrical impedance was assessed with a single-frequency analyzer (BIA-101, 50 kHz; Akern Bioresearch, Florence, Italy). Following ESPEN guidelines [26], participants lay on their backs with legs slightly apart during the examination. They were instructed to refrain from eating, vigorous physical activity, and alcohol consumption for at least 12 h before assessment. After disinfecting the skin with alcohol, electrodes were placed on the dorsal surface of the right hand and the superior surface of the right foot, while sensor electrodes were positioned on the distal right wrist and between the medial and lateral malleoli of the right ankle [27]. Resistance (Rz) and reactance (Xc) were recorded, and body composition parameters—including fat mass (FM), fat-free mass (FFM), total body water (TBW), and extracellular water (ECW)—were derived using specialized software algorithms.

### 2.5. Biochemical Analyses

Fasting blood samples were taken between 8:00 and 9:00 a.m. Serum was then tested for various parameters, including fasting serum glucose (FSG), insulin, triglycerides, total cholesterol, LDL-C, HDL-C, AST, ALT, GGT, uric acid, creatinine, high-sensitivity C-reactive protein, thyroid function, and 25-hydroxyvitamin D. These tests were performed using the COBAS 8000 autoanalyzer (ROCHE Diagnostic SPA, Monza, Italy).

Glycated hemoglobin (HbA1c) was measured using the Capillarys 3 OCTA capillary electrophoresis system (Sebia Italia S.r.l., Bagno a Ripoli, Florence, Italy).

Insulin resistance was estimated using the Homeostasis Model Assessment of Insulin Resistance (HOMA-IR), which was calculated as follows [28]:

HOMA-IR = FSG (mg/dL) × fasting insulin (μIU/mL)/405.

Serum follistatin levels were determined using an ELISA kit (human follistatin ELISA kit, Invitrogen, Vienna, Austria) according to the manufacturer’s instructions. The minimum detectable dose of follistatin was 500 pg/mL.

### 2.6. Variables of Exposure and Confounders

The exposure variable was a plant-enriched diet intervention. To control for potential confounders, five parameters—gender, age, vitamin D, follistatin, and FFM—were incorporated into the final multivariate model, ensuring that the association between the two-month dietary intervention and FM was accurately adjusted for.

### 2.7. Statistical Methods

We conducted a statistical analysis of the baseline variables, presenting continuous data as mean ± standard deviation, median and interquartile range. To compare pre- and post-intervention continuous variables, as the data did not follow a normal distribution, we used the Wilcoxon signed-rank test for paired data. For categorical variables, we used the χ^2^ test to assess differences. Statistical significance was determined using a 95% confidence interval, with *p*-values of 0.05 or less considered statistically significant.

The effect size was calculated using Cohen’s d, which measures the magnitude of the difference between two groups, expressed in standard deviations. Unlike the *p*-value (which indicates only statistical significance), this measure allows us to assess the practical relevance of the results.

Spearman’s rank correlation coefficients and their corresponding *p*-values between the differences before and after the dietary intervention for FM and vitamin D, FM and FFM, and FM and follistatin, were calculated and are displayed in the linear prediction plot.

A Generalized Estimating Equation (GEE) [29] was used to estimate the longitudinal trajectories of FM (pre- and post-plant-enriched diet).

GEE models are used to estimate the average changes in biomarker levels in biomedical research, controlling for covariates and accounting for correlations in response data like repeated measurements within the same subject. Since the outcome variables were not normally distributed, a gamma distribution with an identity link function was employed to model the response, along with an unstructured correlation matrix.

Initially, confounding variables were chosen based on the current literature. The least absolute shrinkage and selection operator (LASSO) was subsequently employed to narrow down the candidate predictors and identify the most relevant ones for model development [30].

Time (pre- vs. post-intervention) was treated as the exposure variable, while gender, age, vitamin D, FFM, and follistatin were treated as covariates.

Three models were constructed to estimate the change in the effect of diet on FM: model *a*: univariate; model *b*: adjusted for gender and age; and model *c*: adjusted for gender, age, vitamin D, FFM, and follistatin.

Stata statistical software version 19.0 (StataCorp 2025, 4905 Lakeway Drive, College Station, TX 77845, USA) was used for the statistical analysis.

## 3. Results

Our sample included an equal number of male and female participants. The average age of women was 45.15 years (±11.46), while that of men was 47.71 years (±9.49). Participants were classified along a spectrum of obesity. The initial mean BMI for women was 36.6 (±4.88), decreasing to 34.74 (±4.55) after two months. The starting BMI for men was 36.79 (±3.65), which dropped to 34.79 (±3.78) following the diet. Table 1 presents the baseline demographic and lifestyle information, and Table 2 displays the group’s parameters before and after the dietary intervention.

Table 2 provides a summary of the entire sample, both before and after the plant-enriched diet. The FibroScan parameters, CAP (used to assess steatosis) and hepatic stiffness (used for fibrosis), both decreased after the diet. By the end of the intervention, most measured parameters showed improvement, especially in fat mass, systolic and diastolic blood pressure, BMI, waist circumference, insulin, HOMA, HbA1c, triglycerides, total and LDL cholesterol, FT3, γ-GT, AST, and ALT, all of which showed significant reductions. Over the two months of dietary treatment, fat mass decreased by an average of 11.8% (±7.02).

The reduction in FM from pre- to post-diet intervention was 4.86 kg, while FFM remained nearly unchanged, with a mean decrease of only 0.64 kg. BMI decreased by 1.93 points (approximately 5%), and waist circumference decreased by 6.34 cm (approximately 5.57%) relative to the initial measurement.

At baseline, patients with a BMI below 35.8 kg/m^2^ (the median) had an average vitamin D level of 27.05 (±9.13), whereas those with a BMI of 35.8 kg/m^2^ or higher had an average level of 21.44 (±5.28).

**Figure 2:**
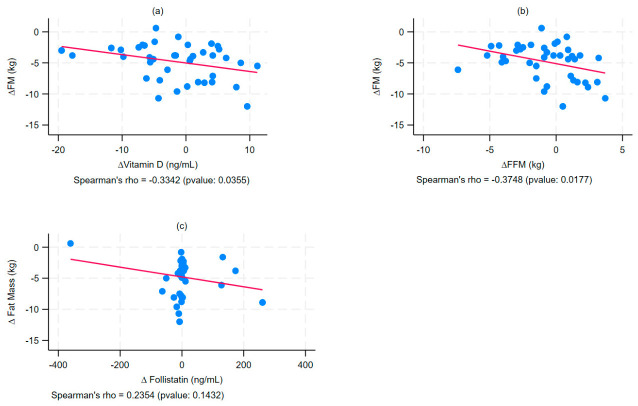
Linear and scatter plots between (**a**) ∆FM and ∆Vitamin D, (**b**) ∆FM and ∆FFM, and (**c**) ∆FM and ∆Follistatin. ∆ = after diet − pre-diet. FM: fat mass, FFM: fat-free mass. The Cartesian coordinates (blue dots) represent the values of the two variables for each observation. The red line identifies the trend of the dots and the type and strength of the correlation between the two variables.

The linear scatter plots (Figure 2) display the inverse proportional relationship between ∆FM (FM post-diet − FM pre-diet) and ∆vitamin D (vitamin D post-diet − vitamin D pre-diet), with a correlation coefficient (rho) of −0.3342 (*p*-value 0.0355). The plots also show the relationship between ∆FM and ∆FFM (FFM post-diet − FFM pre-diet), with a correlation coefficient (rho) of −0.3748 (*p*-value 0.0177), and between ∆FM and ∆follistatin (follistatin post-diet − follistatin pre-diet), with a correlation coefficient (rho) of −0.2354 (*p*-value 0.1432).

Table 3 presents the results of three GEE regression models illustrating the association between a plant-enriched diet and FM in patients with MASLD. A statistically significant association between diet and FM was observed in model a [β = −4.805; *p*-value < 0.001; 95% C.I.: −5.468, −4.143]. Because model a is univariate, the beta regression coefficient, which estimates the mean decrease in FM from baseline to follow-up, reflects the effect of diet alone, without adjusting for potential confounding variables.

When correcting for sex and age, as in model b, the decrease in FM from baseline to follow-up remains almost unchanged, as indicated by the coefficient: β = −4.816 (*p*-value < 0.001). Therefore, age and sex do not further contribute to the decrease in FM from baseline to follow-up.

In the multivariate regression model c, the estimated effect of diet on FM, represented by the beta coefficient, was −5.190 with a *p*-value < 0.001. Accordingly, there was greater reduction in FM at the end of the dietary treatment than in the previous a and b regression models, attributable to the inclusion of vitamin D (β = −0.070, *p*-value: 0.006), follistatin (β = −0.011, *p*-value: <0.001), and FFM (β = −0.275, *p*-value: <0.001), in addition to sex and age (see Table 3).

Model c expands on previous versions by incorporating a wider array of biological covariates, including fat-free mass (FFM) and inflammatory/metabolic markers like follistatin and vitamin D. This allows for a more precise evaluation of the association between the dietary intervention and fat mass by accounting for relevant biological covariates, which are not included in models a and b. The findings suggest that, when accounting for various biomarkers, the intervention was associated with a reduction in fat mass.

## 4. Discussion

The present study demonstrated that a short-term plant-enriched dietary intervention, based on the substitution of one daily carbohydrate portion with *cruciferous* vegetables, was associated with significant improvements in body composition, hepatic steatosis, and metabolic parameters in individuals with obesity and MASLD. These findings support the concept that qualitative dietary modifications, even in the absence of a strictly prescribed caloric restriction, can exert clinically meaningful effects on body composition and metabolic health. A key aspect of this study was that the observed metabolic improvements were attained through simple and pragmatic dietary modifications without any structured caloric restriction. This distinguishes our findings from those of many nutritional interventions in MASLD, which typically rely on energy deficits as the primary driver of metabolic improvement. Although a reduction in caloric intake cannot be entirely ruled out, the lack of a dietary prescription and the real-world design indicate that qualitative dietary changes may independently contribute to the observed effects.

A key finding of this study was the significant reduction in fat mass after only two months of intervention, corresponding to an average relative decrease of approximately 12%, which remained robust after adjustment for multiple confounders, including age, sex, vitamin D, follistatin, and fat-free mass. The finding that such a reduction was achieved through a single-food substitution strategy without explicit caloric prescription reinforces the concept that targeted qualitative dietary changes may represent a minimal yet effective approach to improving adiposity and metabolic health. Given the central role of adiposity in the pathogenesis of MASLD, a 12% reduction in fat mass may have substantially contributed to the observed improvements in hepatic steatosis and metabolic parameters. The observed reduction in fat mass corresponded to an average absolute decrease of approximately 4.9 kg and a relative reduction of nearly 12% over the two-month intervention period. The magnitude of fat mass reduction observed is consistent with previous studies showing that even modest dietary changes can result in clinically relevant improvements in body composition and metabolic risk factors [31]. Importantly, the relatively small decrease in fat-free mass indicates a favorable redistribution of body compartments, which is a critical determinant of metabolic health and long-term weight maintenance [32].

The observed improvement in hepatic steatosis, as reflected by the reduction in CAP values, aligns with existing evidence that dietary modification and weight loss are central to the management of MASLD [5,33]. Previous studies have demonstrated that a weight reduction of 5–10% is associated with significant improvements in liver fat content, inflammation, and fibrosis [6]. The concurrent reductions in CAP, liver stiffness measurements, transaminases, γGT, and insulin resistance suggest a coordinated improvement in hepatic and metabolic function. Clinically, these findings may reflect a reduction in hepatic steatosis and metabolic stress, although the short duration of the intervention limits conclusions regarding fibrosis progression.

These findings reinforce the notion that MASLD is a systemic condition closely linked to metabolic dysfunction rather than an isolated hepatic disorder [34].

A distinctive aspect of this intervention is its focus on food substitution rather than caloric restriction alone. Replacing refined carbohydrates with cruciferous vegetables may reduce the dietary glycemic load and improve postprandial glucose and insulin responses, thereby contributing to the observed decrease in insulin resistance [35]. Diets characterized by high glycemic load have been associated with increased hepatic de novo lipogenesis and fat accumulation, whereas low-glycemic dietary patterns have been shown to improve insulin sensitivity and reduce liver fat accumulation [36].

In addition to glycemic modulation, cruciferous vegetables are rich in dietary fiber, which plays a key role in modulating satiety, energy intake, and gut microbiota composition [37]. Increased fiber intake is associated with reduced body weight, improved lipid metabolism, and decreased systemic inflammation [38]. In addition to fiber, cruciferous vegetables contain glucosinolates and their bioactive metabolites, such as isothiocyanates (e.g., sulforaphane), which have been shown to exert antioxidant and anti-inflammatory effects and regulate lipid metabolism at the molecular level [39,40]. Experimental studies suggest that these compounds can inhibit hepatic lipogenesis, enhance fatty acid oxidation, and reduce oxidative stress, all of which are relevant mechanisms in MASLD pathogenesis [40,41]. Cruciferous vegetables have also been reported to influence gut microbiota composition and microbial metabolite production, which may contribute to improvements in metabolic homeostasis and hepatic function. However, microbiota-related markers were not assessed in the present study, and future investigations incorporating microbiome analyses would help clarify the contribution of gut–liver interactions to the observed effects.

Another relevant finding of this study was the inverse relationship between changes in fat mass and vitamin D levels. Although the overall change in vitamin D was not statistically significant, individuals who experienced greater reductions in fat mass were modestly associated with relative changes in circulating vitamin D levels.

This observation is consistent with previous evidence indicating that vitamin D is sequestered in adipose tissue and that weight loss may increase its circulating bioavailability [42,43]. Furthermore, vitamin D has been implicated in the regulation of adipogenesis, insulin sensitivity, and inflammatory pathways, suggesting that it may play a modulatory role in the metabolic adaptations to weight loss [44].

However, given the observational nature of these analyses and the absence of a control group, these findings should be interpreted cautiously and cannot establish causality.

Follistatin levels also did not change significantly following the intervention, and the correlation with fat mass reduction did not reach statistical significance. However, in the multivariate GEE model, follistatin emerged as a significant predictor of changes in fat mass. Follistatin regulates muscle growth and energy metabolism by inhibiting myostatin and other members of the transforming growth factor-β superfamily [45]. Recent studies have suggested a potential role for follistatin in glucose metabolism, insulin sensitivity, and adipose tissue function [46]. The discrepancy between the lack of significant change in circulating levels and their role in the regression model may reflect complex regulatory mechanisms, including tissue-specific effects or interactions with other metabolic pathways, which warrant further investigation. Although exploratory associations were observed, the absence of significant longitudinal changes in circulating vitamin D and follistatin levels limits mechanistic interpretation. Therefore, these biomarkers should currently be considered exploratory correlates rather than confirmed mediators of the observed metabolic response.

Taken together, these findings support the concept that even minimal dietary modifications, when strategically targeted, may induce clinically meaningful metabolic improvements.

### 4.1. Strengths

This study had several strengths. First, it evaluates a simple, feasible, and easily translatable dietary strategy based on food substitution, which may improve adherence compared with more restrictive dietary interventions. Unlike highly controlled dietary protocols, this approach reflects real-world clinical practice and may facilitate long-term adherence in individuals with metabolic disorders.

Second, the comprehensive assessment of participants—including anthropometric measurements, bioelectrical impedance analysis, biochemical markers, and FibroScan—provides a robust and multidimensional evaluation of metabolic and hepatic outcomes. Third, the inclusion of emerging biomarkers, such as vitamin D and follistatin, adds novelty and contributes to a more integrated understanding of metabolic regulation.

### 4.2. Limitations

Despite these strengths, there are some limitations to consider in this research. The absence of a control group limits causal inference and does not allow exclusion of confounding factors such as spontaneous lifestyle modifications. The relatively small sample size limits the generalisability of the results and may affect the statistical power, especially when detecting changes in biomarkers such as follistatin. In addition, the relatively small sample size in relation to the number of covariates included in the multivariable GEE model may increase the risk of model overfitting; therefore, these analyses should be considered exploratory and interpreted with caution. The short intervention period of two months restricts the ability to make conclusions about long-term adherence or the sustainability of benefits observed. Since total energy intake was not quantitatively assessed, definitive conclusions regarding caloric changes cannot be drawn. It is possible that the substitution of carbohydrate-rich foods with low-energy-density vegetables indirectly reduced overall caloric intake. Therefore, the observed improvements cannot be exclusively attributed to the specific biological properties of cruciferous vegetables. Total caloric intake, dietary composition, glycemic index, fiber intake, and cooking methods were not quantitatively standardized or systematically analyzed during the intervention period. Consequently, spontaneous modifications in dietary patterns beyond the prescribed substitution cannot be excluded. Furthermore, because bioelectrical impedance analysis may be influenced by hydration status, part of the observed changes in body composition could potentially reflect fluid redistribution rather than exclusively changes in adipose tissue.

Nonetheless, this reflects the pragmatic design of the study, aiming to evaluate a real-world, low-complexity intervention rather than a controlled dietary protocol. Finally, while FibroScan is a validated non-invasive method for assessing liver steatosis and fibrosis, it does not match the diagnostic accuracy of a liver biopsy, particularly for evaluating inflammation and early fibrosis stages [5]. Future randomized controlled trials with larger sample sizes and longer follow-ups are needed to confirm these findings.

## 5. Conclusions

In conclusion, this study demonstrates that a short-term plant-enriched dietary intervention, substituting one daily carbohydrate serving with cruciferous vegetables, was associated with a significant reduction in fat mass and improvements in metabolic and hepatic parameters in individuals with obesity and MASLD. These exploratory findings support the potential feasibility and metabolic relevance of simple, sustainable dietary substitution strategies. The observed associations between fat mass, vitamin D, and follistatin suggest that metabolic responses to dietary interventions are influenced by complex biological interactions. Future randomized controlled trials with larger sample sizes and longer follow-ups are needed to confirm these findings and further elucidate the underlying mechanisms.

These findings suggest that meaningful metabolic improvements may be achievable through simple and pragmatic dietary modifications, potentially reducing the need for highly restrictive dietary prescriptions.

## Figures and Tables

**Table 1:** Demographic and lifestyle characteristics at baseline.

Parameter	Value
N	44
Age * (years)	46.34 (10.17)
Gender (%)	
Female	22 (50.00)
Male	22 (50.00)
Smoking habit (%)	
Never	39 (95.12)
Current	2 (4.88)
Physical activity (%)	
<30 min	8 (19.51)
>30 min	26 (63.41)
Sportsman	7 (17.08)
Education (%)	
Secondary school	11 (26.83)
High school	22 (53.66)
Graduated	8 (19.51)
Snoring (%)	
No	10 (24.39)
Yes	31 (75.61)
GERD symptoms (%)	
No	25 (60.98)
Yes	16 (39.02)
Sleepiness (%)	
No	26 (63.41)
Yes	15 (36.59)

* Mean (SD). GERD: gastroesophageal reflux disease.

**Table 2:** Description of the whole sample pre- and post-plant-enriched diet.

Parameters	Pre-Diet	Post-Diet	*p-Value* ^a^	*Effect Size*	
	Mean (SD)	Mean (SD)		*Cohen’s d*	*95% CI*
N	44	44			
**Outcome Variable:**					
Fat mass (kg)	40.85 (10.16)	35.99 (9.37)	<0.001	0.497	0.06; 0.93
**Molecule**					
Follistatin (ng/mL)	43.60 (108.69)	45.30 (80.51)	0.5716	−0.017	−0.43; 0.40
**Ultrasonographic Measures of Liver Steatosis and Fibrosis**					
FibroScan CAP (dB/m)	313.85 (47.04)	278.46 (48.88)	<0.001	0.738	0.28; 1.19
FibroScan LSM (kPa)	7.12 (4.32)	6.41 (3.79)	0.0376		−0.24; 0.59
**Anthropometric and Clinical Parameters**					
SBP (mmHg)	133.10 (12.98)	124.50 (8.61)	<0.001	0.779	0.32; 1.23
DBP (mmHg)	80.61 (11.35)	76.25 (8.14)	<0.001	0.440	0.00; 0.88
PREDIMED questionnaire	8.00 (7.00–9.00)	11.00 (10.00–12.00)	<0.001	−1.689	−2.19; −1.18
BMI (kg/m2)	36.70 (4.24)	34.77 (4.12)	<0.001	0.462	0.02; 0.90
Waist circumference (cm)	113.73 (11.89)	107.39 (12.50)	<0.001	0.519	0.08; 0.96
Fat-free mass (kg)	63.20 (11.65)	62.56 (11.44)	0.0325	0.055	−0.36; 0.47
Body cell mass	35.83 (7.80)	35.57 (7.90)	0.1690	0.033	−0.40; 0.46
**Blood Tests:**					
Glucose (mg/dL)	94.54 (8.91)	94.81 (8.41)	0.5871	−0.031	−0.45; 0.39
Insulin (µIU/mL)	19.44 (10.38)	15.90 (8.39)	<0.001	0.375	−0.05; 0.79
Homa-IR	4.62 (2.70)	3.77 (2.11)	0.0013	0.354	−0.07; 0.77
Hemoglobin A1c	5.48 (0.38)	5.35 (0.33)	<0.001	0.354	−0.07; 0.77
Triglycerides (mg/dL)	123.27 (59.49)	101.53 (54.15)	0.0023	0.382	−0.04; 0.80
Total cholesterol (mg/dL)	192.38 (31.39)	177.34 (29.52)	<0.001	0.494	0.07; 0.91
HDL cholesterol (mg/dL)	49.96 (11.41)	47.24 (10.91)	0.0030	0.243	−0.18; 0.66
LDL cholesterol (mg/dL)	125.80 (28.47)	110.95 (25.20)	<0.001	0.552	0.11; 0.99
AST (U/L)	22.18 (10.70)	19.25 (7.72)	0.0021	0.314	−0.11; 0.73
ALT (U/L)	29.90 (18.05)	22.61 (12.49)	<0.001	0.470	0.04; 0.89
γGT (U/L)	24.57 (14.38)	20.23 (12.11)	<0.001	0.326	−0.09; 0.75
Uric acid (mg/dL)	5.35 (1.73)	5.36 (1.28)	0.2024	−0.004	−0.42; 0.41
Creatinine (mg/dL)	0.86 (0.18)	0.85 (0.16)	0.2040	0.079	−0.34; 0.50
hs-CRP (mg/dL)	0.28 (0.23)	0.40 (0.69)	0.8442	−0.214	−0.65; 0.22
25-hydroxyvitamin D (ng/mL)	24.99 (8.03)	23.69 (4.99)	0.1075	0.354	−0.07; 0.77
TSH (µmU/mL)	1.93 (0.92)	1.87 (1.19)	0.3137	0.049	−0.37; 0.47
FT3 (pg/mL)	3.42 (0.51)	3.14 (0.33)	<0.001	0.647	0.22; 1.07
FT4 (ng/dL)	12.22 (1.21)	11.83 (2.04)	0.0983	0.231	−0.19; 0.65

^a^ Wilcoxon signed-rank test for paired dataLegend: CAP: controlled attenuation parameter; LSM: liver stiffness measurement; SBP: systolic blood pressure; DBP: diastolic blood pressure; PREDIMED: questionnaire; BMI: body mass index; Homa-IR: Homeostasis Model Assessment for Insulin Resistance; HDL: high-density lipoprotein; LDL: low-density lipoprotein; AST: aspartate aminotransferase; ALT: alanine aminotransferase; γGT: gamma-glutamyl transpeptidase; hs-CRP: high-sensitivity C-reactive protein; TSH: thyroid-stimulating hormone; FT4: free tetraiodothyronine; FT3: free triiodothyronine.

**Table 3:** Generalized estimating equation (GEE): expected FM values by time (pre- and post-plant-enriched diet).

FM (kg)	β	*p-Value*	95%CI
Model *a*:			
Pre-diet	0.000		
Post-diet	−4.805	<0.001	−5.468, −4.143
			
Model *b*:			
Pre-diet	0.000		
Post-diet	−4.816	<0.001	−5.487, −4.146
			
Model *c*:			
Pre-diet	0.000		
Post-diet	−5.190	<0.001	−5.721, −4.658
Vitamin D	−0.070	0.006	−0.121, −0.020
Follistatin	−0.011	<0.001	−0.014, −0.008
FFM	−0.275	<0.001	−0.369, −0.182

Model *a*: univariate. Model *b*: adjusted for gender (female vs. male) and age. Model *c*: adjusted for gender (female vs. male), age, vitamin D, FFM, and follistatin; β: regression coefficient; CI: confidence interval.

## Data Availability

The data presented in this study are openly available in https://doi.org/10.6084/m9.figshare.30095326.

## Abbreviations

The following abbreviations are used in this manuscript:

MASLD	Metabolic Dysfunction-Associated Steatotic Liver Disease
MASH	Metabolic Steatohepatitis
CVD	Cardiovascular Diseases
BMI	Body Mass Index
WC	Waist Circumference
CAP	Controlled Attenuation Parameter
LSM	Liver Stiffness Measurement
FSG	Fasting Serum Glucose
LDL-C	Low-Density Lipoprotein Cholesterol
HDL-C	High-Density Lipoprotein Cholesterol
AST	Aspartate Aminotransferase
ALT	Alanine Aminotransferase
γGT	Gamma Glutaminyl Transferase
HbA1c	Glycated hemoglobin
HOMA-IR	Homeostasis Model Assessment of Insulin Resistance
IPAQ	International Physical Activity Questionnaire
PREDIMED	Prevention with Mediterranean Diet
FM	Fat Mass
FFM	Fat-Free Mass
TBW	Total Body Water
